# Construction and validation of nomogram model for prognosis of gastritis patients based on baseline data and inflammatory and infectious markers

**DOI:** 10.3389/fmed.2025.1549901

**Published:** 2025-07-29

**Authors:** Lanfang Zhang, Lu Yang, Lijun Meng, Haiyun Zhang, Yanli Zhu, Fang Yang, Yongmei Qin

**Affiliations:** Department of Gastroenterology, The First Affiliated Hospital of Henan Medical University, Xinxiang, China

**Keywords:** gastritis, inflammatory reactions, infectious pathogens, *Helicobacter pylori*, nomogram model, validation

## Abstract

**Objective:**

Gastritis, a global inflammatory disorder, progresses from symptomatic discomfort to potentially malignant changes. Existing staging systems (e.g., OLGA) focus on cancer risk but ignore modifiable factors like inflammation markers and *Helicobacter pylori* infection. We developed a Nomogram model based on baseline data, inflammatory markers and infectious pathogens for predicting the prognosis of gastritis patients and validating it.

**Methods:**

Retrospectively collect the clinical data of patients diagnosed with gastritis, including baseline characteristics, inflammatory markers, and pathogenic infection test results. Univariate and multivariate analyses were performed to identify independent risk factors associated with the prognosis of gastritis patients, based on which a Nomogram prediction model was constructed. The model’s accuracy, calibration, and discriminative ability were internally validated using the concordance index (C-index), calibration curve, and the area under the receiver operating characteristic curve (AUC).

**Results:**

Among the 185 patients in the training set, 43 (23.24%) had poor treatment outcomes, while in the validation set of 79 patients, 18 (22.78%) exhibited poor treatment outcomes. No statistically significant differences were observed between the training and validation sets in terms of the incidence of poor treatment outcomes, baseline characteristics, or inflammatory and infectious markers parameters (*p* > 0.05). Univariate analysis revealed significant differences (*p* < 0.05) between the poor-outcome and favorable-outcome groups in dietary score, white blood cell count, neutrophil percentage, lymphocyte percentage, C-reactive protein (CRP) level, erythrocyte sedimentation rate (ESR), serum albumin level, and *H. pylori* infection status. Multivariate logistic regression analysis identified dietary score, neutrophil proportion, CRP, ESR, serum albumin level, and *H. pylori* infection as independent risk factors for poor endoscopic treatment outcome (*p* < 0.05). Subsequently, a nomogram prediction model was constructed. The model demonstrated good calibration and fit between predicted and actual outcomes in both the training and validation sets. ROC curve analysis showed that the nomogram model achieved AUC values of 0.808 in the training set and 0.800 in the validation set for predicting gastritis prognosis.

**Conclusion:**

The Nomogram model constructed in this study based on baseline data, inflammation indicators and infectious pathogens can effectively predict the prognosis of patients with gastritis, which can provide a powerful reference for clinical individualized treatment decision-making.

## 1 Introduction

Gastritis, as a common disease of digestive system diseases, is widespread in the world and seriously endangers human health. Its incidence rate is increasing year by year, which brings heavy burden to society and family (1). Gastritis not only leads to epigastric pain, fullness, nausea and vomiting (2–4). Long-term inflammatory stimulation may also cause gastric mucosal atrophy, intestinal metaplasia, and even malignant transformation to gastric cancer, seriously threatening the life safety of patients (5–7). While current systems like OLGA staging stratify risk based on histopathology, they fail to incorporate critical modifiable factors such as baseline characteristics or systemic inflammation, limiting their utility in early intervention (8). In clinical practice, accurately predicting the prognosis of patients with gastritis is of great significance for optimizing treatment strategies and improving the quality of life of patients.

Existing prognostic models predominantly rely on endoscopic or histologic findings, overlooking the synergistic effects of infectious pathogens, host inflammatory responses, and lifestyle factors. As a visual prediction tool based on multi-factor analysis, Nomogram model can effectively integrate multiple variables related to disease prognosis, convert complex mathematical models into intuitive (9). Our nomogram addresses these gaps by unifying three clinically actionable dimensions: (1) host factors (e.g., serum albumin), (2) inflammatory burden (C-reactive protein (CRP), Erythrocyte sedimentation rate (ESR)), and (3) pathogen exposure (*Helicobacter pylori*). This approach aligns with recent calls for multifactorial risk assessment in precision gastroenterology, integrating host, environmental, and microbial factors (10).

The purpose of this study was to make full use of the rich clinical data of gastritis patients diagnosed in our hospital from January 2023 to August 2024, including detailed baseline data, comprehensive inflammation indicators and accurate detection results of infectious pathogens. The Nomogram model was constructed using advanced statistical methods, and the reliability and stability of the model were ensured through strict internal verification, in order to provide a strong support and decision-making basis for the individual treatment of gastritis patients and promote the continuous improvement of the clinical treatment level of gastritis.

## 2 Materials and methods

### 2.1 Study objects

Patients who received gastritis treatment at the hospital between January 2023 and August 2024 were retrospectively enrolled. The 264 patients who were finally included were randomly divided into the training set and the internal split validation according to the ratio of 7:3. All patients were diagnosed with gastritis following the Sydney System criteria (11), with histological confirmation via gastroscopic biopsy specimens. Three key considerations inform our diagnostic approach: First, the Sydney System’s histopathological focus avoids the subjectivity of endoscopic grading. Second, our stringent exclusion of Rome IV-positive functional dyspepsia cases addresses the historical conflation of symptoms with mucosal inflammation. Third, the 40-year literature since *H. pylori*’s discovery confirms that histological changes (e.g., atrophy) have prognostic value independent of symptoms. This study was approved by the Ethics Committee of the First Affiliated Hospital of Xinxiang Medical College, and all patients obtained informed consent.

### 2.2 Inclusion exclusion criteria

#### 2.2.1 Inclusion criteria

(1) Patients who met the diagnostic criteria for gastritis and were confirmed by clinical symptoms, gastroscopy and pathological biopsy; (2) Aged from 18 to 75 years old; (3) Complete clinical data can be provided, including baseline information, inflammation indicators and detection results of infectious pathogens; (4) Patients receiving standardized treatment in our hospital with follow-up time of not less than 12 months.

#### 2.2.2 Exclusion criteria

(1) Suffering from other serious digestive system diseases (Rome IV criteria); (2) Complicated with severe dysfunction of heart, lung, liver, kidney and other important organs or systemic diseases; (3) Recently (within 1 month) used drugs that affect the condition of gastritis or inflammation indicators; (4) Pregnant or lactating women; (5) patients with mental disorders or cognitive dysfunction.

### 2.3 Treatment methods

For the treatment of gastritis patients, mainly take comprehensive treatment. For patients with positive *H. pylori* (Hp), the standard bismuth-based quadruple therapy is firstly adopted, i.e., proton pump inhibitor (such as omeprazole), bismuth-based agent (such as bismuth potassium citrate) and two antibiotics (such as amoxicillin and clarithromycin). The course of treatment lasts for 10 to 14 days, in order to eradicate *H. pylori* and eliminate the important pathogenic factor of infection, thereby alleviating inflammatory reactions. At the same time, mucosal barrier fortifiers (such as hydrotalcite chewable tablets) were administered to form a protective film on the surface of gastric mucosa to reduce the damage of gastric acid and other stimulating factors to gastric mucosa and promote the repair of gastric mucosa. For patients with dyspepsia, gastrointestinal prokinetic agents (such as domperidone) and digestive enzyme preparations (such as compound digestive enzyme capsules) are appropriately used to improve gastrointestinal peristalsis, enhance digestive ability, and relieve abdominal distension, anorexia and other uncomfortable symptoms. In addition, also pay attention to the patient’s diet and lifestyle guidance, suggest patients regular diet, avoid eating spicy, greasy, stimulating food, quit smoking and drinking, keep enough sleep and good state of mind, in order to promote the recovery of gastritis and reduce the risk of recurrence.

### 2.4 Baseline data collection methods

General baseline data, such as age and gender, were mainly collected through a combination of patient medical records and direct patient questioning.

The dietary history questionnaire scores were mainly based on three aspects: First, the type and frequency of food intake (30 points), including the intake of healthy foods such as vegetables, fruits, whole grains, and high-quality protein, as well as the intake of unhealthy foods such as processed meats, sugary beverages, and fried foods; Second, eating behavior (20 points), involving regular eating, slow eating, overeating, excessive dieting, etc.; Third, the eating environment (15 points), covering the stability of meal time and environment, the frequency of eating out, the family dining atmosphere, etc. To evaluate the reliability of the questionnaire, a pre-test was conducted on 30 healthy volunteers, and the overall reliability Cronbach’s *α* was 0.74. Retest was performed on 15 of the volunteers after 2 weeks, and the test–retest reliability coefficient was 0.79. Inter-rater consistency was evaluated by two nutritionists independently scoring 25 randomly selected questionnaires, with an intraclass correlation coefficient (ICC) of 0.83. These results support the reliability of the questionnaire for institutional use, although future studies may adopt standardized tools such as the Food Frequency Questionnaire (FFQ) or 24-h recall for cross-comparison and broader validation.

### 2.5 Detection methods of inflammatory markers

All inflammatory markers were tested and samples were obtained from venous blood collection. The medical staff would select the vein at the elbow of the patient’s upper limb as the blood collection site. The skin was disinfected with iodophor or alcohol cotton balls first, and then the disposable blood collection needle was used to quickly puncture the vein to allow blood to flow into the tube along the vacuum blood collection tube connected with the blood collection needle. The blood collection amount is determined according to the specific requirements of the test items. In general, for the indicators such as white blood cell count, neutrophil proportion, lymphocyte proportion, C-reactive protein, erythrocyte sedimentation rate, serum albumin level and procalcitonin, the blood collection amount of about 3–5 mL can meet the test requirements. After completion of blood collection, the blood collection tube was gently inverted several times to fully mix the blood with the anticoagulant to ensure the quality of the sample, and then the blood collection tube was sent to the laboratory for testing in time.

The count and classification of white blood cells rely on blood cell analyzer to count and distinguish the proportion of various white blood cells such as neutrophils and lymphocytes according to the size, morphology and internal structure characteristics of white blood cells using techniques such as electrical impedance and light scattering. C-reactive protein detection uses immunoturbidimetry. When the C-reactive protein in serum combines with the specific antibody in the reagent, it will change the turbidity of the reaction system. The instrument determines the content of C-reactive protein by detecting this change and comparing it with the standard substance. For erythrocyte sedimentation rate test, the anticoagulant was placed vertically in a erythrocyte sedimentation tube to observe the sedimentation rate of red blood cells within a certain time. The faster the sedimentation rate was, the more active the inflammation would be. Detection of serum albumin level use biochemical analyzer to calculate that content of albumin by detect absorbance after the albumin is colored by specific chemical reaction; Procalcitonin is detected by chemiluminescent immunoassay using the light signal intensity generated by antigen–antibody binding reaction and compared with the standard curve, to achieve the accurate determination of procalcitonin concentration.

### 2.6 Infection pathogen infection rate detection method

#### 2.6.1 Gastroscopy

After the patient had fasted for 6–8 h, local anesthesia was applied to his throat, followed by slow oral insertion of a gastroscope into the esophagus, stomach and duodenum. During operation, a doctor carefully observes the morphology, color and luster of gastric mucosa through the observation channel of the gastroscope. For the suspicious lesion sites found, such as mucosal swelling, erosion, ulcer, and the like, a small amount of gastric mucosa tissue, generally two to four pieces, is accurately clamped by a biopsy forceps, and the obtained tissue is put into a specimen bottle filled with a specific preservation solution for subsequent pathological examination of *H. pylori*, rapid urease test, bacterial culture, smear, culture, pathological examination of gastric fungi, and the like, so as to determine whether *H. pylori* infection and gastric fungal pathogen infection exist.

#### 2.6.2 Venous blood collection

The thick and straight vein at the elbow part of the patient’s upper limb was selected. First, the skin was disinfected with iodine in the range of about 5–6 cm in diameter. After the iodine was completely dried, the medical staff quickly penetrated the vein using a disposable blood collection needle at an angle of 15–30 degrees and collected 3–5 mL of blood into a vacuum blood collection tube containing an anticoagulant. After collection, the blood collection tube was gently reversed 4 to 5 times to allow the blood to be fully mixed with the anticoagulant. After these blood samples are centrifuged to obtain serum or plasma, the specific antibodies against EB virus and cytomegalovirus can be detected by serological detection methods such as enzyme-linked immunosorbent assay (ELISA) to determine the stage of infection; The presence of nucleic acids of Epstein–Barr virus, cytomegalovirus and enterovirus is confirmed by using nucleic acid detection techniques such as real-time fluorescent quantitative PCR to diagnose the infection status of these viruses.

### 2.7 Treatment prognosis judgment method

Imaging Findings: Complete resolution of inflammatory exudative changes in the gastric mucosa, normalization of gastric wall thickness, and no new ulcers or space-occupying lesions were observed (assessed via standardized endoscopic scoring). Endoscopy: The gastric mucosa was evaluated using the Los Angeles (LA) classification for erosive gastritis: Grade 0: No mucosal breaks, Grade A: One or more mucosal breaks ≤5 mm, Grade B: One or more mucosal breaks >5 mm without confluence, Grade C: Confluent mucosal breaks involving <75% of the gastric fold circumference, Grade D: Confluent mucosal breaks involving ≥75% of the gastric fold circumference, Histopathology: Inflammatory cell infiltration was graded based on the updated Sydney System criteria: Neutrophil activity: 0 (none) to 3 (severe, >10 cells/high-power field), Chronic inflammation: 0 (none) to 4 (severe, dense lymphoid aggregates), Atrophy: 0 (none) to 4 (severe, extensive gland loss), Clinical Symptom Score: A validated 4-point Likert scale (0 = none, 3 = severe) was used to assess: Epigastric pain, Bloating, Nausea, Vomiting, Composite Prognostic Score (0–14 points): Endoscopic LA grade (0–4 points), Histological inflammation score (0–10 points, sum of neutrophil activity, chronic inflammation, atrophy), Symptom score (0–4 points, sum of four symptoms), Good Prognosis: Composite score ≤4 points, Poor Prognosis: Composite score >4 points.

### 2.8 Statistical methods

SPSS26.0 software was used and Chi-square test was used to count the data. The t value was substituted into the measurement data, and *p* < 0.05 showed statistical significance.

## 3 Results

### 3.1 Treatment outcomes, baseline data, inflammatory and infectious markers parameters comparison between training set and verification set

Among the 185 patients in the training set, 43 (23.24%) had poor treatment outcomes, while among the 79 patients in the validation set, 18 (22.78%) had poor treatment outcomes. There was no statistically significant difference in the incidence of poor treatment outcomes, baseline data, and inflammatory and infectious markers parameters between the training and validation sets (*p* > 0.05), as shown in Table 1.

**Table 1:** Training and verification set baseline data, inflammatory and infectious markers comparison.

Project		Training set (*n* = 185)	Validation set (*n* = 79)	Statistical values	*p*-value
Age (years)		55.21 ± 2.34	57.68 ± 2.13	1.534	0.126
Gender	Man	102	45	0.074	0.784
	Woman	83	34		
BMI (kg/m^2^)		23.56 ± 3.21	23.89 ± 3.56	0.740	0.460
Dietary assessment score		42.83 ± 3.26	42.59 ± 3.57	0.532	0.595
Smoking history	Yes	44	28	3.794	0.051
	No	141	51		
Drinking history	Yes	59	25	0.002	0.961
	No	126	54		
Hypertension	Yes	63	18	3.305	0.069
	No	122	61		
Diabetes	Yes	64	18	3.605	0.057
	No	121	61		
Cardiovascular disease	Yes	56	15	3.584	0.058
	No	129	64		
White blood cell count (×10/L)		6.52 ± 1.23	6.85 ± 1.56	1.836	0.067
Neutrophil proportion (%)		65.23 ± 8.15	66.05 ± 8.32	0.744	0.457
Proportion of lymphocytes (%)		25.32 ± 5.15	26.05 ± 5.32	1.044	0.297
C-reactive protein (mg/L)		12.56 ± 5.23	13.12 ± 5.56	0.747	0.455
Erythrocyte sedimentation rate (mm/h)		15.32 ± 6.15	16.05 ± 6.32	0.875	0.381
Serum albumin level (g/L)		40.56 ± 3.23	41.12 ± 3.56	1.251	0.212
Procalcitonin (ng/mL)		0.12 ± 0.05	0.13 ± 0.06	1.399	0.162
*Helicobacter pylori*	Positive	103	42	0.141	0.707
	Negative	82	37		
Epstein–Barr virus	Positive	38	14	0.278	0.597
	Negative	147	65		
Cytomegalovirus	Positive	28	11	0.064	0.799
	Negative	157	68		
Enterovirus	Positive	19	9	0.073	0.786
	Negative	166	70		
Fungal pathogen infection	Positive	27	8	0.932	0.334
	Negative	159	71		

### 3.2 Baseline data, inflammatory and infectious markers parameters comparison between the poor-prognostic group and the good group

The results of univariate analysis showed that there were significant differences in dietary score, white blood cell count, neutrophil proportion, lymphocyte proportion, C-reactive protein, erythrocyte sedimentation rate, serum albumin level and *Helicobacter pylori* infection between the two groups (*p* < 0.05) (see Table 2).

**Table 2:** Comparison of baseline data, inflammatory and infectious markers parameters between the poor-prognostic group and the good-prognostic group.

Project		Group with poor prognosis (*n* = 43)	Good prognosis group (*n* = 142)	Statistical values	*P*-value
Age (years)		56.78 ± 7.25	57.46 ± 6.41	0.747	0.455
Gender	Man	25	77	0.204	0.651
	Woman	18	65		
BMI (kg/m^2^)		26.83 ± 3.52	25.96 ± 3.27	1.501	0.135
Dietary assessment score		41.57 ± 3.21	43.24 ± 3.26	2.953	0.004
Smoking history	Yes	12	33	0.390	0.532
	No	31	109		
Drinking history	Yes	30	96	0.071	0.789
	No	13	46		
Hypertension	Yes	26	74	0.927	0.335
	No	17	68		
Diabetes	Yes	19	45	2.277	0.131
	No	24	97		
Cardiovascular disease	Yes	18	38	3.565	0.059
	No	25	104		
White blood cell count (×10/L)		8.67 ± 1.38	8.02 ± 1.25	2.915	0.004
Neutrophil proportion (%)		73.86 ± 8.35	69.28 ± 7.64	3.369	0.001
Proportion of lymphocytes (%)		23.58 ± 5.35	26.37 ± 5.21	3.057	0.003
C-reactive protein (mg/L)		11.87 ± 2.65	10.84 ± 2.06	2.678	0.008
Erythrocyte sedimentation rate (mm/h)		22.84 ± 3.67	20.73 ± 3.85	3.272	0.001
Serum albumin level (g/L)		32.45 ± 4.12	34.58 ± 4.03	3.021	0.003
Procalcitonin (ng/mL)		0.16 ± 0.06	0.15 ± 0.03	1.473	0.142
*Helicobacter pylori*	Positive	34	69	12.423	0.001
	Negative	9	73		
Epstein–Barr virus	Positive	10	28	3.462	0.063
	Negative	33	114		
Cytomegalovirus	Positive	8	11	3.126	0.077
	Negative	35	131		
Enterovirus	Positive	9	18	1.854	0.173
	Negative	34	125		
Fungal pathogen infection	Positive	6	13	0.386	0.534
	Negative	37	129		

### 3.3 Analysis of prognostic risk factors in patients with gastritis

The prognosis was regarded as the dependent variable (0 = good, 1 = poor), and the factor with *p* < 0.05 in univariate analysis was regarded as the covariate. Further multivariate Logistic regression analysis showed that Dietary assessment score, neutrophil proportion, C-reactive protein, erythrocyte sedimentation rate, serum albumin level, and *H. pylori* were the independent risk factors for poor prognosis after endoscopic treatment (*p* < 0.05), To evaluate the interaction between co exposure of pathogens, *H. pylori* and EB virus were added to a multifactorial model. The results showed that the interaction term between *H. pylori* and EB virus was not statistically significant (*p* > 0.05), indicating that there was no significant synergistic or additive effect between the two in affecting the prognosis of gastritis. In the regression model, all variables demonstrated acceptable collinearity thresholds: tolerance values >0.1, variance inflation factors (VIF) < 10, and condition indices <30. Furthermore, no covariance decomposition revealed multiple variables with variance proportions >50% under the same eigenvalue. These findings collectively indicate the absence of multicollinearity among covariates, as shown in Table 3.

**Table 3:** Logistic regression analysis of prognostic risk factors in patients with gastritis.

Factor	*B*	S. E.	Wald	*P*	OR	95%CI
Dietary assessment score	−0.198	0.067	8.761	0.003	0.821	0.720–0.935
Neutrophil proportion	0.094	0.029	10.261	0.001	1.099	1.037–1.164
C reactive protein	0.208	0.094	4.919	0.027	1.232	1.02511.481
Erythrocyte sedimentation	0.207	0.061	11.405	0.001	1.230	1.091–1.387
Serum albumin level	−0.154	0.053	8.455	0.004	0.857	0.773–0.951
*Helicobacter pylori*	1.627	0.517	9.891	0.002	5.088	1.846–14.023
EBV by *Helicobacter pylori*	−0.630	0.526	1.435	0.231	0.533	0.190–1.493

### 3.4 Gastritis patients prognosis nomogram prediction model

**Figure 1:**
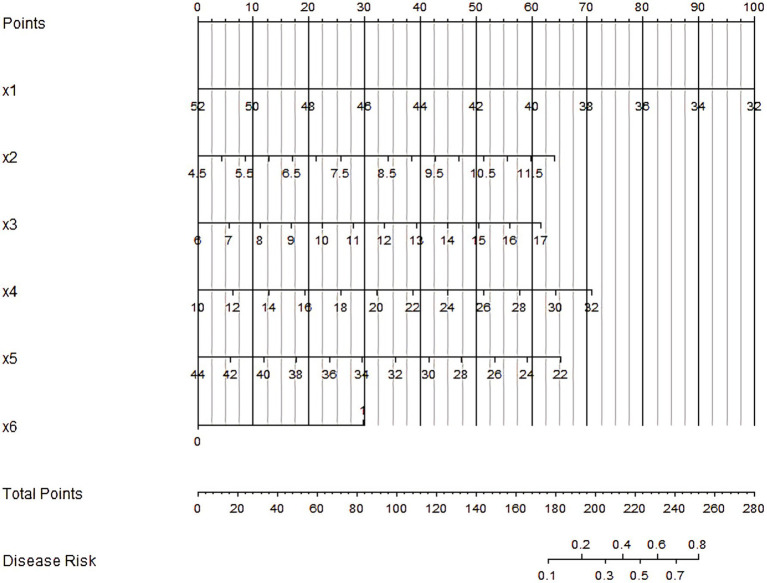
Nomogram of prognostic nomogram prediction model for gastritis patient. X1–X6 are respectively: Dietary assessment score, Neutrophil proportion, C-reactive protein, Erythrocyte sedimentation rate, Serum albumin level, *Helicobacter pylori*.

Based on the independent risk factors identified by multivariate Logistic regression analysis, the nomogram prediction model for the prognosis of gastritis patients was constructed. Each independent risk factor in the model was scored, and the total score for predicting the prognosis of gastritis patients was calculated, which was reflected in the incidence of poor prognosis of gastritis patients. The higher the total score was, the higher the accuracy of prognosis for gastritis patients was (see Figure 1).

### 3.5 Assessment and validation of prognostic nomogram prediction model for gastritis patients

**Figure 2:**
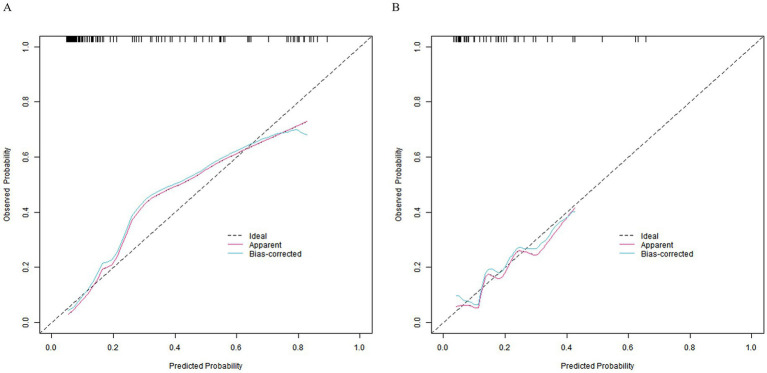
Calibration curves. Panel **(A)** represents the calibration curve of the training set, and **(B)** represents the calibration curve of the validation set.

**Figure 3:**
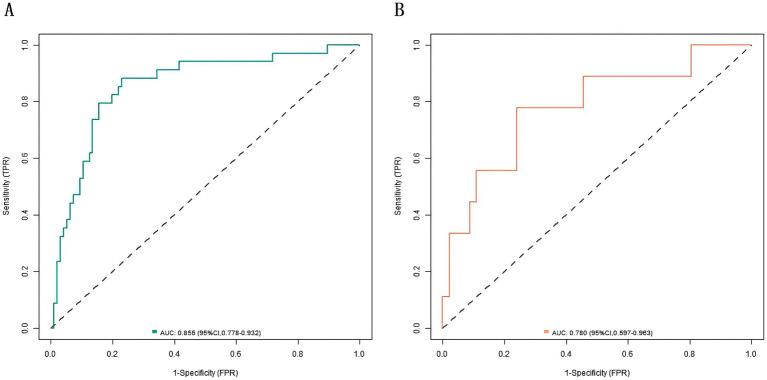
Receiver operating characteristic (ROC) curves. **(A)** ROC curve of the training set. **(B)** ROC curve of the validation set.

In the training and validation sets, the nomogram model C-index was 0.803 and 0.791, respectively, the calibration curve showed the mean absolute errors of predicted and actual values were 0.143 and 0.108, respectively, and the Hosmer-Lemeshow test results were *χ*^2^ = 11.025, *p* = 0.200 and *χ*^2^ = 7.839, *p* = 0.449, respectively. The ROC curves were displayed in the training set and the verification set. The AUC of the nomogram model for predicting the prognosis of patients with gastritis was 0.808 (95% CI: 0.721–0.896) and 0.800 (95% CI: 0.589–1.000), respectively. The sensitivity and specificity were 0.765, 0.844, and 0.778, 0.826, respectively. The calibration curves are shown in Figure 2 and the ROC curves are shown in Figure 3.

### 3.6 Analysis of decision curve of prognostic nomogram nomogram prediction model for gastritis patients

**Figure 4:**
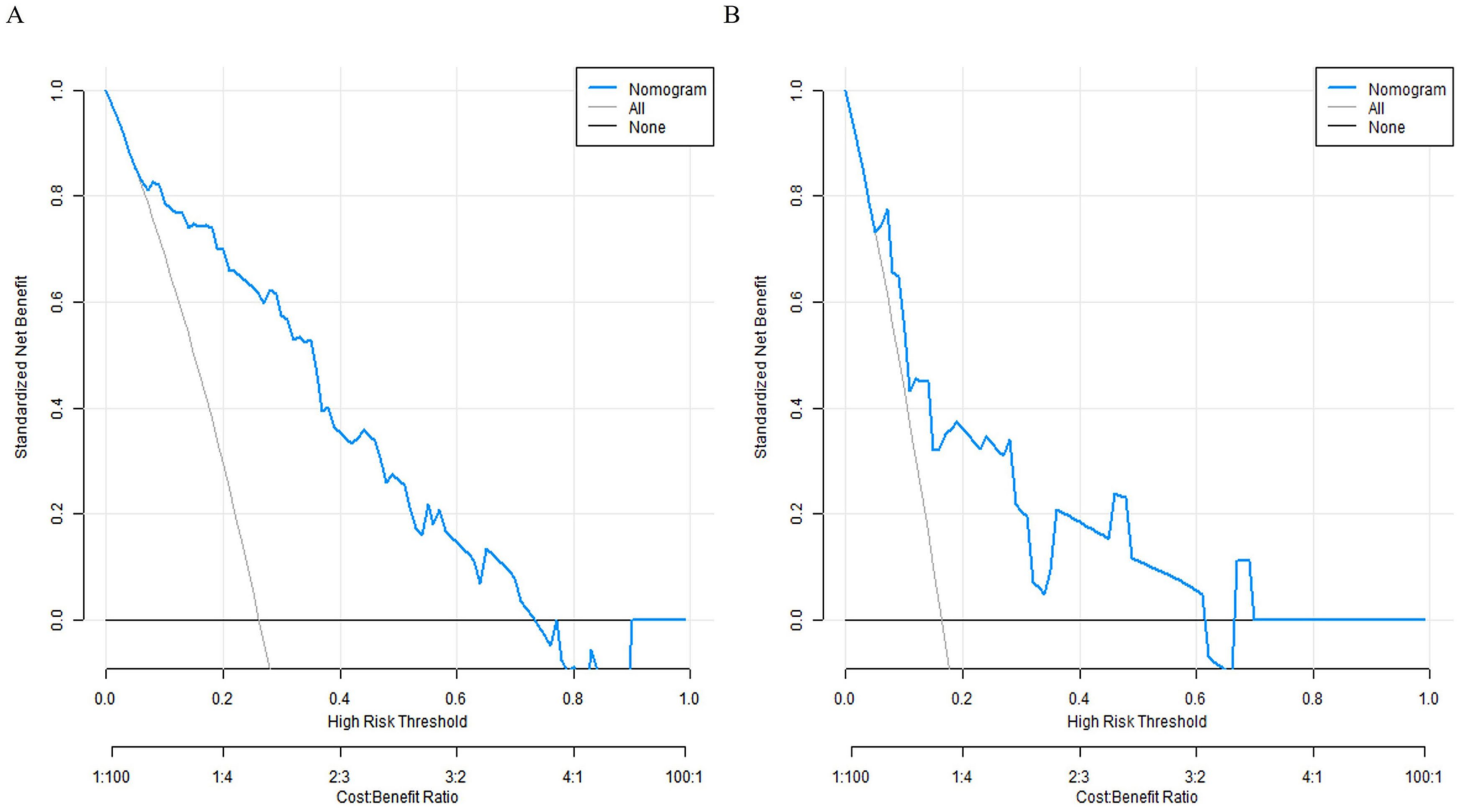
Decision curves. **(A)** Decision curve for the training set. **(B)** Decision curve for the validation set.

The decision curve showed that when the threshold probability was about 0. 05–0. 95, the nomogram model constructed in this study would have more clinical benefits in predicting the prognosis of patients with gastritis (see Figure 4).

## 4 Discussion

The pathogenesis of gastritis involves multiple dimensions, from abnormal cell signaling in molecular biology to macro-level lifestyle, environmental factors and the interaction of pathogens and hosts (12, 13). With the continuous advancement of the concept of precision medicine, the diagnosis and treatment of gastritis are no longer limited to the traditional symptom judgment and single index detection, but more attention is paid to the comprehensive, comprehensive and individual evaluation model. At the cellular level, the infiltration of inflammatory cells and the imbalance between damage and repair of gastric mucosal epithelial cells are finely regulated by a variety of cytokines and growth factors (14, 15). In the macro human environment, factors such as dietary habits, psychological pressure, and the types and loads of infectious pathogens are intertwined to jointly affect the occurrence, development and prognosis of gastritis (16). This complex disease ecosystem urgently needs a prognostic prediction tool that can integrate multiple information.

In this study, we explored the clinical data of 112 patients with gastritis, and successfully constructed and verified the value of Nomogram model based on baseline data, inflammation indicators and infectious pathogens in the prognosis prediction of gastritis. In the initial comparison between the training set (78 cases) and the validation set (34 cases), there were no significant differences in baseline data, inflammatory and infectious markers parameters, and the incidence of poor treatment prognosis, so as to ensure the randomization and balance of subgroups. The dietary scores were different between the poor prognosis group and the good prognosis group, and the poor dietary habits, such as excessive intake of high salt, high fat, and high glucose foods, will destroy the normal physiological environment of gastric mucosa, affect gastric acid secretion and gastrointestinal peristalsis rhythm, and weaken the barrier function of gastric mucosa, thus providing a hotbed for the development of inflammation and seriously affecting the prognosis (17, 18). Among the inflammatory indicators, the changes in white blood cell count, C-reactive protein and erythrocyte sedimentation rate profoundly reflect the key role of inflammatory response in the prognosis of gastritis. An increase in white blood cell count means the aggravation of inflammatory reaction. While resisting pathogens, the accumulation of a large number of inflammatory cells may also cause excessive immune damage to gastric mucosa. The released inflammatory mediators will further destroy the integrity of gastric mucosa, form a vicious circle, and aggravate the condition (19). As sensitive indicators of systemic inflammation, C-reactive protein and erythrocyte sedimentation rate increase to indicate the broad and strong degree of inflammatory response. The persistent high inflammatory state seriously interferes with the normal metabolism and physiological function of the body, and hinders the recovery process of gastritis (20). Serum albumin levels were significantly reduced in the group with poor prognosis, which is a comprehensive reflection of metabolic disorder and gastrointestinal nutrition absorption disorder in the inflammatory state. Hypoproteinemia not only affects the overall nutritional status of the body, but also weakens the activity of immune cells and the material basis required for tissue repair, making it difficult for gastric mucosa to obtain adequate nutritional support for repair, and greatly affects the prognosis (21). *Helicobacter pylori* infection is more prominent in the group with poor prognosis. As an important pathogenic factor of gastritis, it continuously releases toxins and enzymes, destroys the gastric mucosal barrier, and triggers chronic inflammation, which increases the risk of malignant transformation of gastric mucosal epithelial cells under the long-term effect, and seriously interferes with the prognosis trajectory of gastritis (22, 23).

The Nomogram model built based on these significantly different factors performed well in both the training set and the validation set. The C-index was 0.858 and 0.777, respectively. The calibration curve closely fitted the actual situation with small average absolute error. The result of Hosmer–Lemeshow test was good. The area under the ROC curve was higher than 0.7 in both groups, and the sensitivity and specificity were excellent. This fully proves the high precision and reliability of the model in predicting the prognosis of patients with gastritis, and it can provide valuable decision support for clinicians to develop personalized treatment strategies for patients with different prognostic risks, such as strengthening anti-inflammatory treatment for high-risk patients, optimizing *H. pylori* eradication plan and stricter diet management, which is expected to significantly improve the prognosis of patients. This study has the following limitations: First, as a single-center retrospective study, the sample size is relatively limited (*n* = 264), and the proportion of poor outcomes in the validation set of 79 patients is 22.78%. The sample size and the incidence of adverse events may affect the statistical power, and the universality of the model for external populations may be limited. Second, although the self-reported dietary scoring system in this study has undergone internal consistency testing (Cronbach’s *α* = 0.74), test–retest reliability verification (0.79) and inter-rater verification (ICC = 0.83), there may still be recall bias (such as overestimating the intake of healthy foods or underestimating the intake of high salt). Future studies should integrate standardized assessment tools (such as FFQ, 24-h dietary recall) or objective markers (such as urinary sodium excretion) to reduce subjective bias. In addition, the current model is only constructed based on baseline data, and dynamic indicators such as dynamic changes in CRP during treatment (such as the decrease in CRP after 2 weeks of treatment), fluctuations in serum albumin, or improvement in symptom scores are not included, which may limit its ability to dynamically predict the prognosis. Finally, although this study tested the interaction between *H. pylori* and Epstein–Barr virus and cytomegalovirus in the multivariate analysis, due to sample size limitations, it failed to comprehensively evaluate the impact of combined exposure to other pathogens (such as *Helicobacter pylori* + enterovirus). Future studies should expand the sample size and systematically evaluate the impact of interactions of various pathogens on the prognosis.

The nomogram prediction model developed in this study integrates baseline characteristics, inflammatory markers, and pathogenic infection data to achieve accurate prognosis prediction for gastritis patients, providing an important tool for individualized clinical management. The model’s high predictive performance demonstrates the advantage of multifactorial assessment, though further refinement through multicenter studies and external validation remains necessary. Future research should focus on incorporating novel biomarkers, expanding multicenter data collection, and integrating patient-reported outcomes to advance gastritis prognosis evaluation toward precision and personalized medicine, ultimately improving clinical outcomes and reducing gastric cancer risk.

## Data Availability

The raw data supporting the conclusions of this article will be made available by the authors, without undue reservation.
